# Publisher Correction: Southern Ocean in-situ temperature trends over 25 years emerge from interannual variability

**DOI:** 10.1038/s41467-021-22318-6

**Published:** 2021-03-17

**Authors:** Matthis Auger, Rosemary Morrow, Elodie Kestenare, Jean-Baptiste Sallée, Rebecca Cowley

**Affiliations:** 1 Sorbonne Université, CNRS, LOCEAN, Paris, France; 2 CNES, Toulouse, France; 3 LEGOS, CNRS/IRD/CNES/University of Toulouse III, Toulouse, France; 4 CSIRO Marine and Atmospheric Research, Hobart, Tasmania Australia

**Keywords:** Climate-change impacts, Ocean sciences, Physical oceanography

Correction to: *Nature Communications* 10.1038/s41467-020-20781-1, published online 21 January 2021.

The original version of this Article contained an error in Fig. 2 that inverted the order of the zones. This has been corrected in both the PDF and HTML versions of the Article.

