# Supplementary material for: Southern Ocean in-situ temperature trends over 25 years emerge from interannual variability
Source: Nat Commun. 2021 Jan 21;12:514. doi: 10.1038/s41467-020-20781-1 (PMC7819991; doi:10.1038/s41467-020-20781-1)
Supplement: Supplementary file 1 — Supplementary Information [file 41467_2020_20781_MOESM1_ESM.pdf]

Supplementary Information for Southern Ocean *in-situ* temperature trends over 25 years emerge from interannual variability by Auger et al.

Supplementary Note 1: Definition of Winter Water Upstream Zone:

In our study, Winter Water is defined as the water-mass with a temperature lower than 2°C between 54°S and 61.5°S. To find the winter origin of this Winter Water along the mean SURVOSTRAL line near 140°E, we used backward-flowing Lagrangian trajectories derived from surface altimetric currents on the first day of November, December, January and February between 1993 and 2003. We found that 90% of SURVOSTRAL WW particles at 140°E could be located within the surface mixed layer upstream in the previous winter (May to August) in the region centered at 57 - 61°S and 119°E and 146°E.

Supplementary Note 2: Zone A Interannual variations in SST and Subtropical water extent

Supplementary Figure 1b shows how the extent of subtropical waters crossing our section (waters warmer than 11°C) varies in direct relation to the interannual upper ocean temperature changes in Zone A (correlation r=0.58).

Cool anomalies in the Subantarctic zone were noted in SURVOSTRAL data during 1994-1995, due to the persistence of cold-core eddies near 140°E separated from the SAF (Morrow et al., 2004), whereas the 1996 cool anomalies are influenced by a weaker input of STW from the Tasman Sea (Supplementary Figure 1b; Morrow and Kestenare 2014). Stronger warming events in 2001-2002 and in 2014-2016 have anomalously warm SST (Supplementary Figure 1a). These years may be influenced by incursions of subtropical waters carried by the Tasman Sea extension south of Tasmania following large La Nina events, impacting the extent of STW crossing 140°E, as well as more warm-core eddies propagating through the region (Pilo et al., 2015; Morrow and Kestenare, 2014).

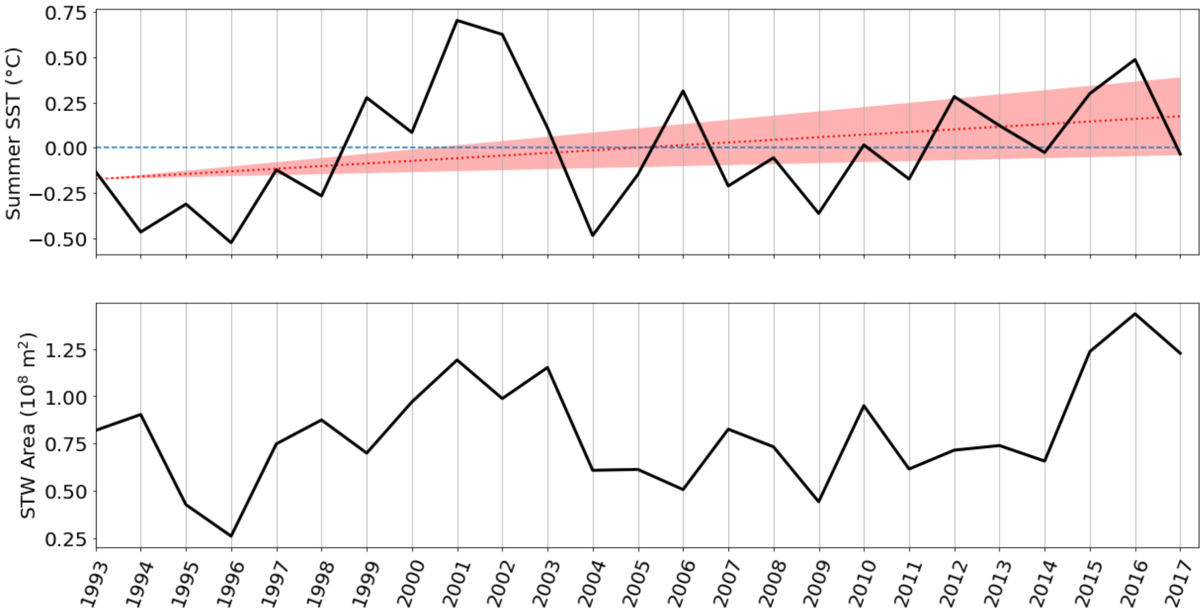

Supplementary Figure 1: Interannual variations in Sea Surface Temperature and Subtropical water extent. a. NDJF Mean sea surface anomalies on the SURVOSTRAL line within zone A bounds. b. Subtropical Water area within the transect.

Supplementary Note 3: Austral summer mean temperature sections

Mean temperature sections are calculated for each austral summer, from November to February (NDJF). A monthly mean section is calculated for each month and for each year, and the four monthly values of NDJF are then averaged to obtain the austral summer temperature section. This reconstruction was chosen since there are certain years with more missing data in one month than another (Supplementary Figure 5). The available profiles for each year are impacted by the distinct seasonal changes seen in Supplementary Figure 3. For example, a simple austral summer average of all available profiles but with points missing in November would show an annual warming compared to the average NDJF conditions. To reduce the effects of this sampling bias, we replace the missing values at each point in the monthly section with the 25-yr monthly mean value. Note that this step is not applied for the anomalies.

Supplementary Figure 2 shows the austral summer mean temperature sections for each year from 1993 to 2017. Black regions have no data available for the entire austral summer, which occurred during the early voyages in 1992-1993 as the XBT system was being established, and south of 66°S. Hatched regions have only 2 out of 4 months with good observations, and the other months are filled with the 25-year mean monthly value.

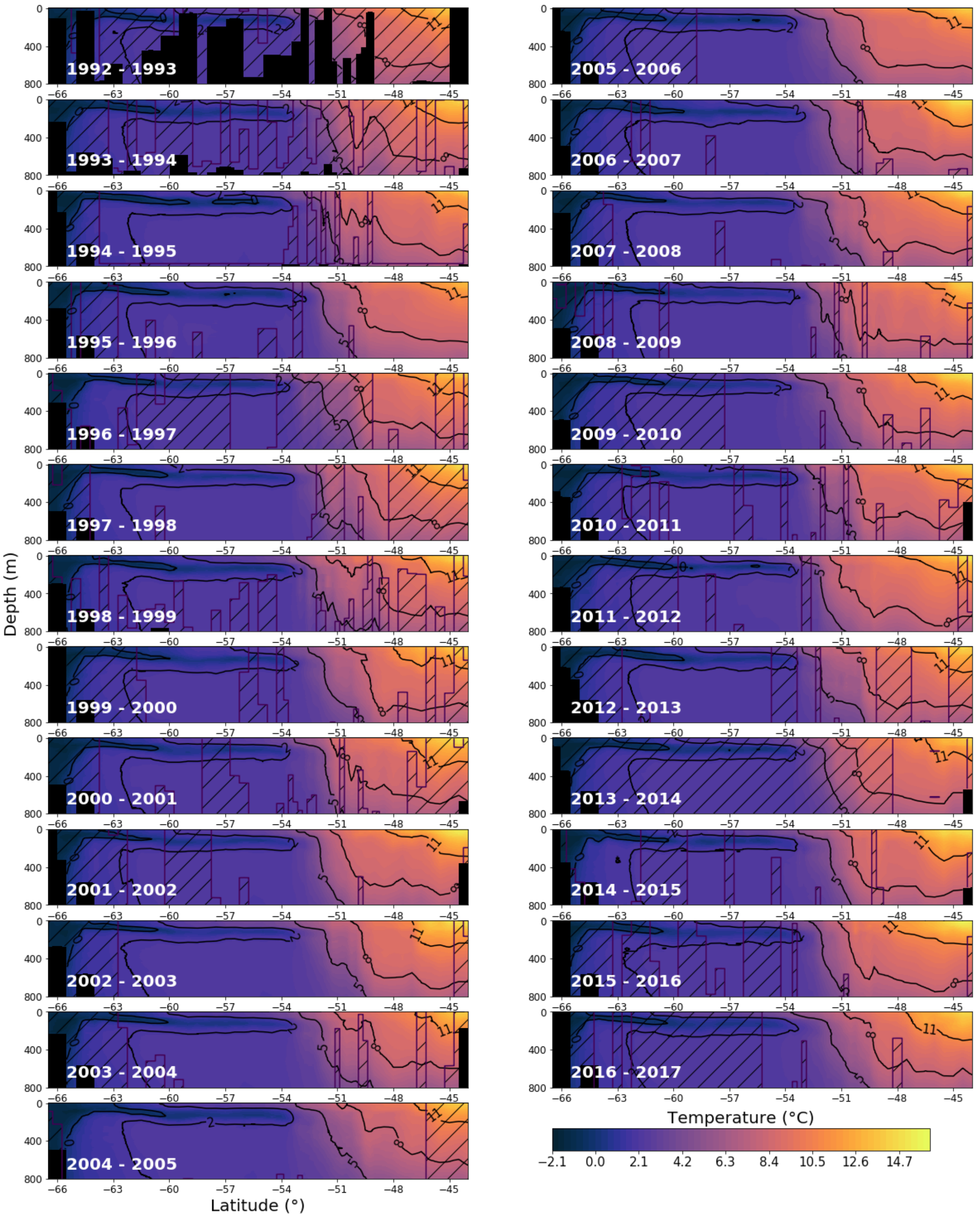

Supplementary Figure 2: Austral summer mean temperature transects. Austral summer mean temperature structure from 1993 to 2017 based on SURVOSTRAL profiles from the months NDJF. Hatched areas are where climatological monthly mean had to be added at least

twice to compute the summer mean. Black contours show the mean isotherms of 0, 2, 5, 8 and 11°C.

Supplementary Note 4: Calculating monthly mean temperature sections

Monthly mean XBT temperature sections are computed between October and March from the 25 years of Austral Summer data (Supplementary Figure 3), following a similar method to that used in Morrow et al. (2008). XBT profiles are associated to a latitude box and interpolated linearly on the depth range of the study grid (see Methods), then all profiles available each month are averaged within a latitude-depth grid box.

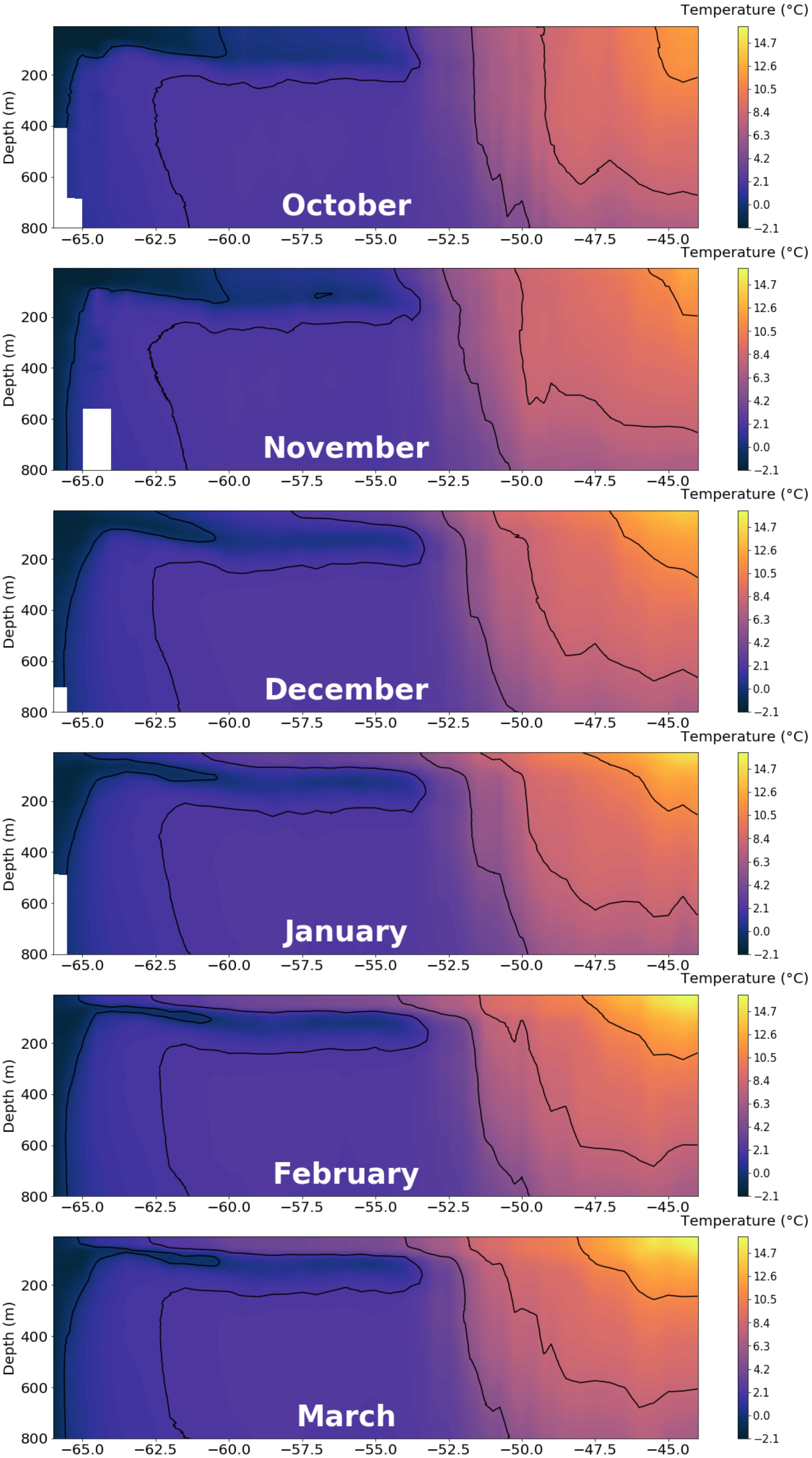
Temperature (°C)
14.7
12.6
10.5
8.4
6.3
4.2
2.1
0.0
−2.1
Depth (m)
200
400
600
800
−65.0
−62.5
−60.0
−57.5
−55.0
−52.5
−50.0
−47.5
−45.0
October
November
December
January
February
March

Supplementary Figure 3: Monthly temperature climatology. October to March monthly mean values based on SURVOSTRAL data from 1993 to 2017. Black contours show the mean isotherms of 0, 2, 5, 8 and 11°C.

Supplementary Note 5: Number of measurements for each grid point

The distribution of the number of measurements per grid box over the 25-years (Supplementary Figure 4) highlights the larger number of observations in the Northern part of the section; seasonal sea-ice is present in the region south of 62°S, particularly in October and November.

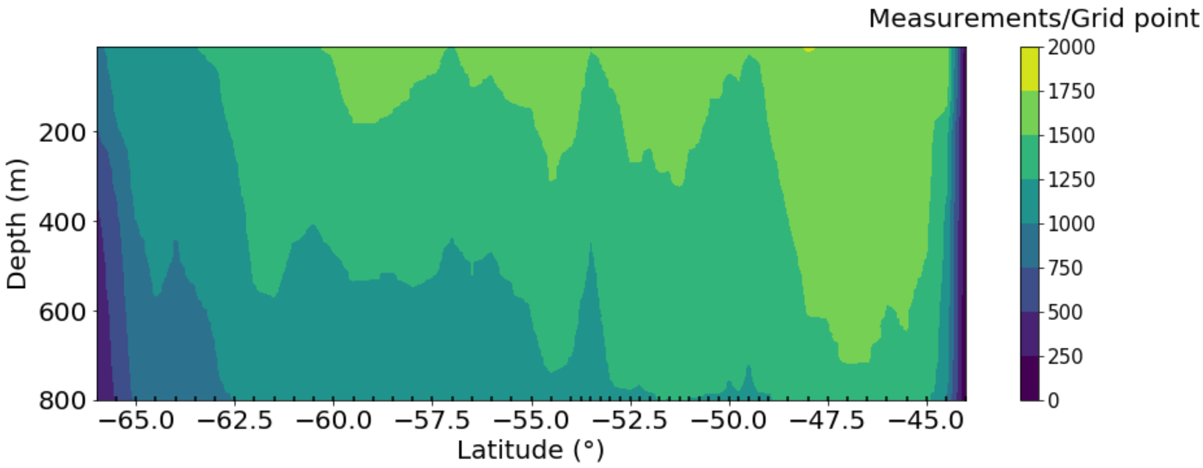

Supplementary Figure 4: Number of measurements per grid point for the 25 years of SURVOSTRAL XBT data. Black dots on the x-axis shows the size of the grid points. The increase of resolution in the polar frontal zone between 49°S and 54°S is compensated by an increase in XBT sampling frequency in this zone.

Supplementary Note 6: Temporal and Spatial repartition of XBT profiles

Supplementary Figure 5 shows the number of XBT profiles used in the analysis over the 25-year period, with valid data reaching at least 200 m (Note our analysis is based on

measurements up to 800m). The number of profiles varies by month (Supplementary Figure 5, left). Profiles are collected every 2h during the ship's route (roughly 35 km separation), doubling to 1h sampling in the polar frontal zone (18 km separation) from 49-54°S.

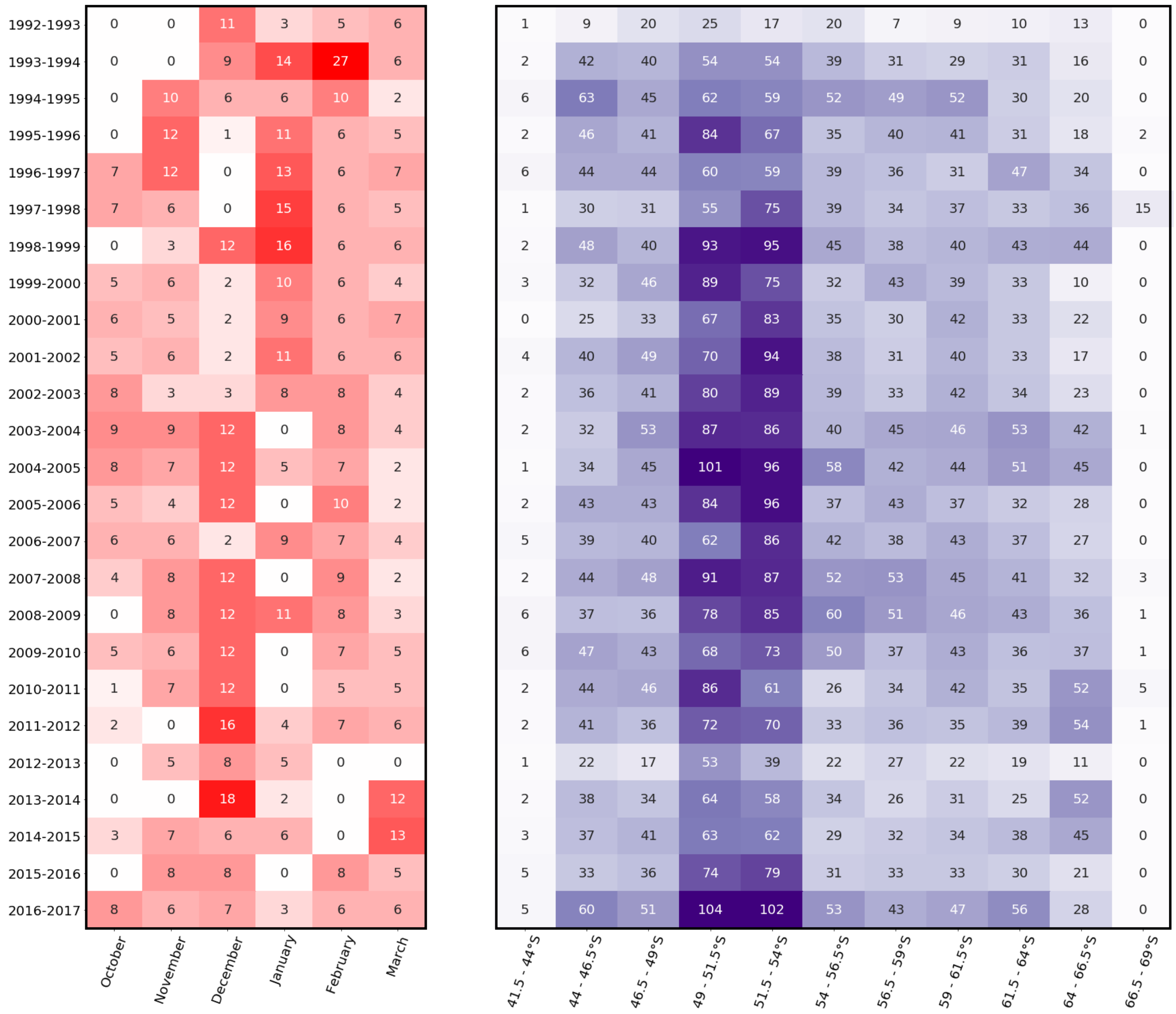

Supplementary Figure 5. Temporal and Spatial repartition of XBT profiles. For each sampling season: a. Number of days with at least one profile sampled within each month. b. Repartition of XBT profiles by latitude band.

Supplementary Note 7: Gridding and measurement error estimation

The measurement error estimation is made by computing the standard deviation (Supplementary Figure 6) of multiple profiles from the same transect occurring within the same latitude-depth box. A standard deviation is computed only when there are at least 3 data points within the same grid point for each transect. This standard deviation is averaged over all transects on the full grid, in order to represent the gridding and measurement error estimation.

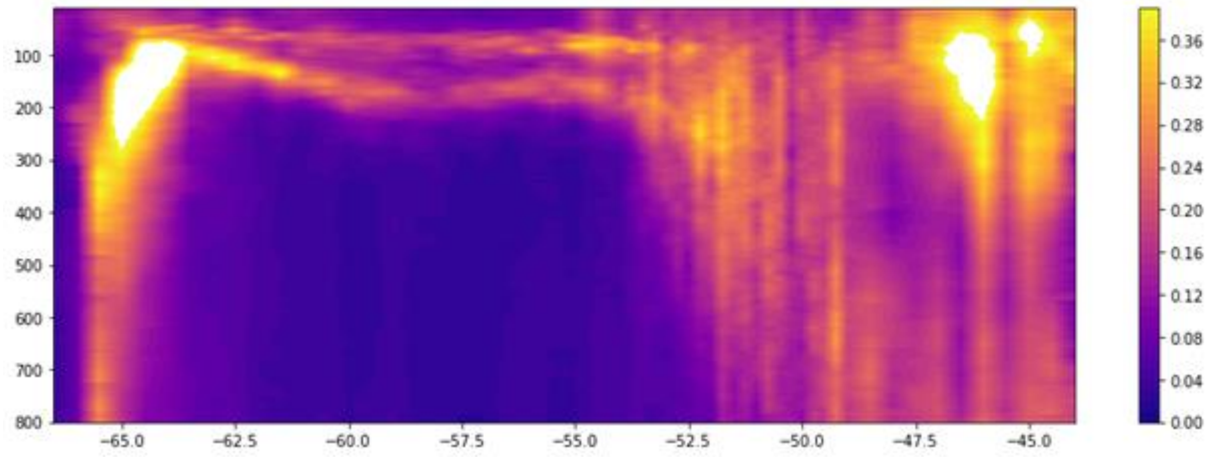

Supplementary Figure 6. Gridding and measurement error estimation. Mean standard deviation of measurements sampled on the same grid point for one transect, based on all transects

Supplementary Note 8: XBT data processing and selection along the SURVOSTRAL line

XBT profiles were processed twice : 1) using the official UNESCO fall-rate processing (Hanawa et al 1995 [55]) available on the IMOS website (http://thredds.aodn.org.au/thredds/catalog/IMOS/SOOP/SOOP-XBT/DELAYED/Line_IX28_Dumont-d-Urville-Hobart/catalog.html.) and 2) with the more recent fall rate and depth correction (Cheng et al 2014 [52]) as recommended by the SOOP XBT panel (http://thredds.aodn.org.au/thredds/catalog/IMOS/SOOP/SOOP-

XBT/PRODUCTS/BiasCorrectedData_ChengEtAl_2014/Line_IX28_Dumont-d-Urville-Hobart/catalog.html). Our trend results over the last 25-years across the Southern Ocean are similar when using either fall-rate correction. This reprocessing allowed us to show the stability and robustness of our results.

XBT profiles with the most recent processing[52] are selected based on their distance to the mean path of the transect. In order to avoid sampling regions with different dynamics, we remove profiles located more than 3° longitude away from the mean path, or samples east of 142°E and south of 65.5°S. Over the 10238 vertical profile sampled, 614 have been discarded due to their location.

SURVOSTRAL campaigns have DOI : 10.18142/172;
